# Stress-induced antinociception to noxious heat requires α_1A_-adrenaline receptors of spinal inhibitory neurons in mice

**DOI:** 10.1186/s13041-021-00895-3

**Published:** 2022-01-03

**Authors:** Sawako Uchiyama, Kohei Yoshihara, Riku Kawanabe, Izuho Hatada, Keisuke Koga, Makoto Tsuda

**Affiliations:** 1 Department of Molecular and System Pharmacology, Graduate School of Pharmaceutical Sciences, Kyushu University, 3-1-1 Maidashi, Higashi-ku, Fukuoka, 812-8582 Japan; 2 Laboratory of Genome Science, Biosignal Genome Resource Center, Institute for Molecular and Cellular Regulation, Gunma University, 3-39-15 Showa-machi, Maebashi, Gunma 371-8512 Japan; 3 Department of Neurophysiology, Hyogo College of Medicine, Nishinomiya, Hyogo 663-8501 Japan

**Keywords:** Stress-induced antinociception, α_1A_-adrenaline receptor, Spinal dorsal horn, Inhibitory interneurons, Mouse

## Abstract

It is well known that acute exposure to physical stress produces a transient antinociceptive effect (called stress-induced analgesia [SIA]). One proposed mechanism for SIA involves noradrenaline (NA) in the central nervous system. NA has been reported to activate inhibitory neurons in the spinal dorsal horn (SDH), but its in vivo role in SIA remains unknown. In this study, we found that an antinociceptive effect on noxious heat after acute exposure to restraint stress was impaired in mice with a conditional knockout of α_1A_-adrenaline receptors (α_1A_-ARs) in inhibitory neurons (*Vgat-Cre*;*Adra1a*^flox/flox^ mice). A similar reduction was also observed in mice treated with *N*-(2-chloroethyl)-*N*-ethyl-2-bromobenzylamine, a selective neurotoxin for NAergic neurons in the locus coeruleus (LC). Furthermore, whole-cell patch-clamp recordings using spinal cord slices revealed that NA-induced increase in the frequency of spontaneous inhibitory postsynaptic currents in the substantia gelatinosa neurons was suppressed by silodosin, an α_1A_-AR antagonist, and by conditional knockout of α_1A_-ARs in inhibitory neurons. Moreover, under unstressed conditions, the antinociceptive effects of intrathecal NA and phenylephrine on noxious heat were lost in *Vgat-Cre*;*Adra1a*^flox/flox^ mice. Our findings suggest that activation of α_1A_-ARs in SDH inhibitory neurons, presumably via LC-NAergic neurons, is necessary for SIA to noxious heat.

**Supplementary Information:**

The online version contains supplementary material available at 10.1186/s13041-021-00895-3.

It has long been known that acute exposure to stress reduces behavioral responses to noxious stimuli in rodents and humans [1], a phenomenon generally known as stress-induced analgesia (SIA). Studies using experimental models of stress have proposed that acute physical stress (for example, foot shock, forced swimming, and restraint) leads to the activation of descending pain inhibitory pathways via neurotransmitters and neuropeptides, and subsequently produces a transient antinociceptive effect [1]. The locus coeruleus (LC)-noradrenergic (NAergic) neurons are among these descending pathways [1]. Previous studies have shown that LC-NAergic neurons are activated by acute exposure to physical stress [2]. Spinal NA acts on adrenaline receptors (ARs). It has been reported that α_2_-ARs are expressed on the terminals of primary afferent nociceptive neurons and inhibit glutamate release [3]. Furthermore, the stimulation of α_1_-ARs enhances the frequency of spontaneous inhibitory currents (sIPSCs) in the spinal substantia gelatinosa (SG) neurons [4]. Moreover, a recent single-cell analysis of SDH neurons has revealed a predominant expression of α_1_-ARs in inhibitory neurons [5]. However, it remains unknown whether α_1_-ARs in inhibitory neurons play a role in the control of nociceptive behavior by spinal NA and acute stress exposure because an experimental tool that enables selective manipulation of the expression or function of α_1_-ARs in inhibitory neurons has not been developed until recently.

To investigate this, we utilized the recently developed mouse line *Adra1a*^flox/flox^ mice [6] and generated mice with conditional knockout (cKO) of α_1A_-ARs in inhibitory interneurons by crossing *Vgat*-*Cre* mice (Cre expression under the control of vesicular γ-aminobutyric acid [GABA] transporters) (see Additional file 1). RNAscope in situ hybridization confirmed that *Adra1a* mRNA expression was almost absent in *Vgat*^+^ (*Slc32a1*) SDH neurons of *Vgat-Cre*;*Adra1a*^flox/flox^ mice (Additional file 2). Consistent with previous studies [7], a transient increase in the latency to produce nociceptive behavioral responses in the hot-plate test was observed in control mice (*Adra1a*^flox/flox^ mice) after exposure to acute restraint stress (Fig. 1a), a well-known model of SIA [1]. In contrast, stress-induced antinociception to noxious heat was impaired in *Vgat*-*Cre*;*Adra1a*^flox/flox^ mice (Fig. 1a). Since acute restraint stress has been shown to activate LC-NAergic neurons [2], we examined the involvement of descending LC-NAergic signaling using *N*-(2-chloroethyl)-*N*-ethyl-2-bromobenzylamine (DSP-4), a selective neurotoxin for the LC-NAergic neurons [6, 8]; DSP-4-treated wild-type (WT) mice also showed a reduction in the restraint stress-induced antinociceptive effect on noxious heat (Fig. 1b).

**Fig. 1:**
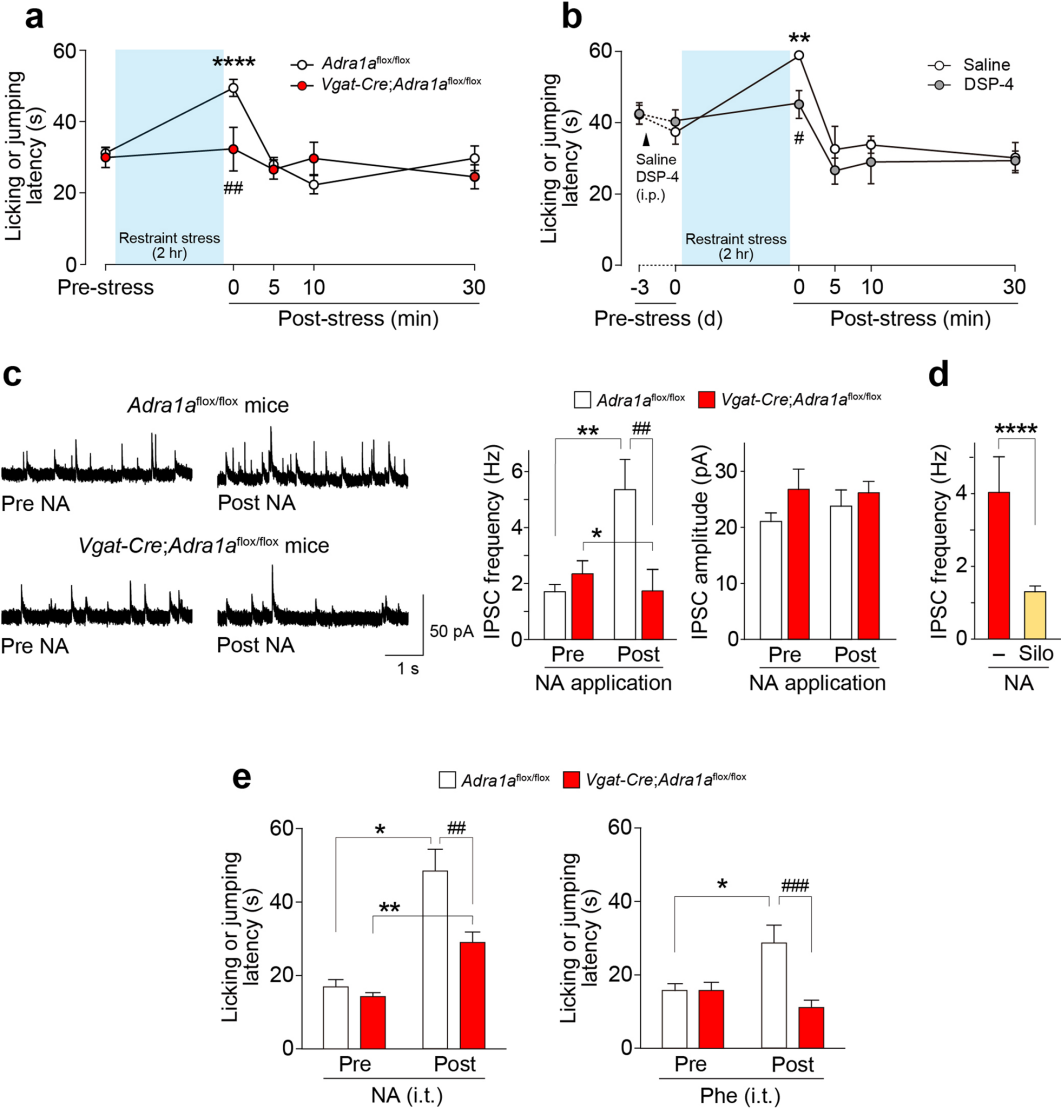
α_1A_-ARs in spinal inhibitory neurons contribute to acute restraint stress-induced antinociception to noxious heat. **a**,** b** Latency to evoke nociceptive behaviors (licking or jumping) by noxious heat stimulation (hot-plate test) following acute restraint stress for 2 h in *Adra1a*^flox/flox^ mice (n = 9) and *Vgat*-*Cre*;*Adra1a*^flox/flox^ mice (n = 8) (**a**), and WT mice pretreated intraperitoneally with saline (n = 10) or DSP-4 (n = 10) (**b**). **P < 0.01 and ****P < 0.0001 vs. Pre-stress, ^#^P < 0.05 and ^##^P < 0.01 vs. saline-treated mice and *Adra1a*^flox/flox^ mice at post-stress (0 min), respectively. **c** Frequency and amplitude of sIPSCs in SG neurons in spinal cord slices from *Adra1a*^flox/flox^ mice or *Vgat*-*Cre*;*Adra1a*^flox/flox^ mice before (Pre NA) and after NA (20 μM) application (Post NA) (n = 14–15 neurons). *P < 0.05, **P < 0.01, ^##^P < 0.01.** d** Effect of silodosin (Silo: 40 nM) on NA-induced facilitation of sIPSC frequency in SG neurons (n = 9–10 neurons). ****P < 0.0001.** e** Latency to evoke nociceptive behaviors (licking or jumping) in hot-plate test at 10 min after intrathecal administration of NA (10 nmol, n = 7) or phenylephrine (Phe: 50 nmol, n = 8) in *Adra1a*^flox/flox^ mice or *Vgat*-*Cre*;*Adra1a*^flox/flox^ mice, *P < 0.05, **P < 0.01, ^##^P < 0.01, and ^###^P < 0.001. Data show the mean ± SEM

These results raise the possibility that NA signals via α_1A_-ARs in SDH inhibitory neurons are crucial for acute restraint stress-induced antinociception. To examine whether NA activates SDH inhibitory neurons via α_1A_-ARs, we performed whole-cell patch-clamp recordings using spinal cord slices and measured the frequency and amplitude of sIPSCs in SG neurons. Consistent with the findings of previous studies [4], the application of NA to spinal slices from *Adra1a*^flox/flox^ mice increased the frequency, but not amplitude, of sIPSCs in SG neurons (Fig. 1c). The sIPSC frequency was not increased (even slightly decreased) in the *Vgat*-*Cre*;*Adra1a*^flox/flox^ mice. Similarly, acute blockade of α_1A_-ARs by silodosin, an α_1A_-AR-specific antagonist, inhibited the NA-induced increase in sIPSC frequency (Fig. 1d). These results indicate that NA activates *Vgat*^+^ inhibitory neurons in the SDH via α_1A_-ARs.

The intrathecal administration of NA is known to produce antinociceptive effects against noxious heat [9]. Thus, we predicted that α_1A_-ARs in inhibitory neurons are involved in the antinociceptive effects of spinal NA. We found that the intrathecal NA-induced antinociceptive effect on noxious heat was attenuated in *Vgat*-*Cre*;*Adra1a*^flox/flox^ mice (Fig. 1e). A striking inhibition was observed in the antinociceptive effect of the α_1_-AR-selective agonist phenylephrine administered intrathecally. Thus, these data suggest that activation of α_1A_-ARs in spinal *Vgat*^+^ inhibitory neurons is necessary for the antinociceptive effect of spinal NA on noxious heat.

SIA is a well-known phenomenon in animals and humans as a biological response of organisms to noxious stimuli [1]; however, its underlying mechanism remains to be fully understood. By utilizing the recently developed mouse line *Adra1a*^flox/flox^ mice [6], which enables cKO of α_1A_-ARs in a cell-type-specific manner, this study demonstrated for the first time the crucial role of α_1A_-ARs in spinal inhibitory neurons in acute restraint stress-induced antinociceptive response to noxious heat in mice. The α_1A_-ARs in inhibitory neurons can be activated by NA released from the descending LC-NAergic neurons. This is supported by the result of our and other studies showing (1) the activation of LC-NAergic neurons by acute exposure to physical stress [2], (2) the attenuation of stress-induced antinociception by DSP-4, (3) the expression of α_1A_-ARs predominantly in inhibitory neurons [5], and (4) a failure of NA to facilitate inhibitory synaptic inputs to SG neurons in mice lacking α_1A_-ARs. Furthermore, the intrathecal administration of α_1_-AR agonist failed to induce antinociception by α_1A_-AR-cKO in *Vgat*^+^ inhibitory neurons. Together, these results suggest that acute restraint stress could activate descending LC-NAergic neurons and subsequently α_1A_-ARs in *Vgat*^+^ inhibitory SDH neurons, which in turn suppresses spinal nociceptive transmission evoked by noxious heat, although our data do not exclude a possible involvement of α_1A_-ARs in the brain (as *Vgat*-*Cre*;*Adra1a*^flox/flox^ mice also lack α_1A_-AR expression in the brain).

While this study highlighted the role of α_1A_-AR in SIA to noxious heat, it should be noted that other ARs, especially α_2_-AR, are also involved in the regulation of nociceptive transmission in the SDH. It has been shown that NA and clonidine, an α_2_-AR agonist, hyperpolarize SG neurons [10] and reduce excitatory synaptic inputs on SG neurons by inhibiting presynaptic glutamate release from primary afferents [3]. Behaviorally, the antinociceptive response to noxious heat to the tail (tail-flick test) evoked by forcing the rats to swim in cold water, an acute stress model, is potentiated by intraperitoneal injection with an α_2_-AR agonist [11]. A global KO of α_2_-ARs suppresses noise stress-induced thermal hypoalgesia in the hot-plate test [12]. The involvement of α_2_-AR could be predicted in our present data showing that the antinociception by intrathecal NA was reduced but not abolished in *Vgat*^+^ neuron-selective α_1A_-AR-KO mice in which phenylephrine antinociception was not produced. Thus, it is conceivable that the extent of the contribution of each α-AR subtype may be determined by the nature, duration, and intensity of the stressor and behavioral assays for antinociceptive responses; in the case of thermal antinociception after acute restraint stress, the role of α_1A_-ARs expressed on *Vgat*^+^ inhibitory neurons in the SDH could be predominant.

In the control of spinal nociceptive information processing and transmission by NA, we have previously demonstrated that the activation of α_1A_-ARs in *Hes5*^+^ SDH astrocytes causes hypersensitivity to a light mechanical stimulus in mice [6]. A similar hypersensitivity is also induced by the intrathecal administration of NA and phenylephrine [6]. The different effects of these agonists (antinociception to noxious heat vs. pronociceptive to light mechanical force) could be dependent on both their doses for intrathecal administration and their selective modulation of the somatosensory modality. It has been demonstrated that low doses of intrathecal NA (0.03 and 0.1 nmol) and phenylephrine (0.015 and 0.05 nmol) selectively enhance behavioral sensitivity to mechanical stimuli without affecting heat [6]. On the contrary, at a relatively higher dose, NA and phenylephrine begin to produce antinociceptive effects on noxious heat (as seen in this and previous studies [6]) and interestingly have little, if any, effect on behavioral responses to mechanical stimuli [6]. Given that α_1A_-ARs in *Hes5*^+^ astrocytes and *Vgat*^+^ inhibitory neurons in the SDH are required for modulating behavioral responses to mechanical and heat stimuli at low and high doses, respectively, of intrathecal NA and phenylephrine, the different dose-dependent effects of NA and phenylephrine could be related to their actions on the different cell types. Supporting this notion, the chemogenetic activation of *Hes5*^+^ astrocytes selectively enhances behavioral sensitivity to mechanical stimuli but not thermal stimuli [6]. However, the reason for the disappearance of mechanical hypersensitivity at a high dose of NA and phenylephrine and the role of *Vgat*^+^ inhibitory neurons in such disappearance remains unclear. This may be associated with an interaction between *Vgat*^+^ inhibitory neurons and *Hes5*^+^ astrocytes in the SDH, which are important subjects for future investigations.

## Supplementary Information

**Additional file 1:** Materials and methods.

**Additional file 2: **Supplementary Figure 1.

## Data Availability

All data generated or analyzed during this study are included in this published article and its Additional file.

## Abbreviations

AR: Adrenaline receptor
DSP-4: *N*-(2-Chloroethyl)-*N*-ethyl-2-bromobenzylamine
LC: Locus coeruleus
NA: Noradrenaline
SDH: Spinal dorsal horn
SG: Substantia gelatinosa
SIA: Stress-induced analgesia
sIPSCs: Spontaneous inhibitory postsynaptic currents
WT: Wild-type
