# Supplementary material for: Stress-induced antinociception to noxious heat requires α1A-adrenaline receptors of spinal inhibitory neurons in mice
Source: Mol Brain. 2022 Jan 3;15:6. doi: 10.1186/s13041-021-00895-3 (PMC8721982; doi:10.1186/s13041-021-00895-3)
Supplement: Supplementary file 1 — Additional file 1: Materials and methods. [file 13041_2021_895_MOESM1_ESM.docx]

**Additional file 1**

**Materials and methods**

**Animals**

Male C57BL/6J mice (CLEA Japan), and male and female *Vgat-Ires-Cre* mice (Stock No: 016962, The Jackson Laboratory) [1] and *Adra1a*^flox/flox^ mice [2][1] were used. The genetic background of *Adra1a*^flox/flox^ mice was derived from BDF1 [(C57BL/6 × DBA/2)F1] strain, and in this study we used *Adra1a*^flox/flox^ mice that were backcrossed one time onto the C57BL/6 background. To generate *Vgat-Cre*;*Adra1a*^flox/flox^ mice, *Adra1a*^flox/flox^ mice were crossed with *Vgat-Ires-Cre* (*Vgat-Cre*) mice, and the obtained *Vgat-Cre*;*Adra1a*^flox/+^ mice were further crossed with *Adra1a*^flox/flox^ mice. *Adra1a*^flox/flox^ littermate mice were used as controls. All mice used were 8–12 weeks old at the start of each experiment and were housed at 22 ± 1 °C with a 12-h light–dark cycle with food and water ad libitum. All animal experiments were conducted according to relevant national and international guidelines contained in the ‘Act on Welfare and Management of Animals’ (Ministry of Environment of Japan) and ‘Regulation of Laboratory Animals’ (Kyushu University) and under the protocols approved by the Institutional Animal Care and Use committee review panels at Kyushu University.

**Restraint stress model**

Mice were anesthetized with acute isoflurane inhalation for 30 sec. These mice were put into a 50-mL plastic conical tube (Falcon, Corning, USA) with air wholes on the top, and were restraint for 2 hr according to a previously described method [3]. Immediately after restraint stress, mice were placed on a hot-plate (50 ℃).

**Hot-plate test**

The latency to evoke nociceptive behaviors (licking or jumping) against heat stimulation (50 ℃ for SIA [3], and 52 ℃ for non-SIA) was recorded. A cut-off time was set at 60 sec to avoid tissue damage.

**Whole-cell patch-clamp recordings**

According to our previously described method [4], mice were deeply anesthetized with urethane (1.2–1.5 mg/kg, i.p.), and the lumbar spinal cord was removed and placed in a cold high-sucrose artificial cerebrospinal fluid (sucrose aCSF; 250 mM sucrose, 2.5 mM KCl, 2 mM CaCl_2_, 2 mM MgCl_2_, 1.2 mM NaH_2_PO_4_, 25 mM NaHCO_3_, and 11 mM glucose). A parasagittal spinal cord slice (250–300 μm thick) was made with a vibrating microtome (VT1200; Leica, Wetzlar, Germany), and then the slices were kept in oxygenated aCSF solution (125 mM NaCl, 2.5 mM KCl, 2 mM CaCl_2_, 1 mM MgCl_2_, 1.25 mM NaH_2_PO_4_, 26 mM NaHCO_3_, and 20 mM glucose) at room temperature (22–25°C) for at least 30 min. The spinal cord slice was then put into a recording chamber, where it was continuously superfused with aCSF solution at 25–28°C at a flow rate of 4–6 mL/min. Patch pipettes were filled with an internal solution (120 mM CsMeSO_4_, 15 mM CsCl, 10 mM HEPES, 5 mM QX-314, 4 mM MgATP, 0.3 mM Na_2_GTP, 0.2 mM EGTA, 10 mM TEA-Cl and 8 mM NaCl [pH 7.28] adjusted with CsOH), and whole-cell patch-clamp recordings were made from SG neurons. Recordings were made with the Axopatch 700B amplifier and pCLAMP 10.4 acquisition software (Molecular Devices). Data were digitized with an analog-to-digital converter (Digidata 1550; Molecular Devices), stored on a personal computer with a data acquisition program (ClampeX version 10.4; Molecular Devices), and analyzed with a software package (Clampfit version 10.4; Molecular Devices). Spontaneous inhibitory postsynaptic currents (sIPSCs) were recorded in the voltage-clamp mode at a holding potential of 0 mV. The drugs used were L-norepinephrine hydrochloride (NA, 20 μM; Sigma) and silodosin (40 nM; Wako). All drugs were dissolved in aCSF solution. NA was superfused for 2 min, and silodosin was applied 5 min before NA application. The frequency and amplitude of sIPSCs for 1 min of pre- and post-NA application were quantified using Minianalysis software (Synapsoft).

**Drug administration**

DSP-4 (50 mg/kg, Sigma) [5,6] was intraperitoneally injected 3 days before the exposure to restraint stress. L-Norepinephrine hydrochloride (10 nmol in 5 μL saline; Sigma) and (R)-(-)-phenylephrine hydrochloride (50 nmol in 5 μL saline; Wako) were intrathecally injected 10 min before the hot-plate test. Intrathecal injection was performed using a 25-μL Hamilton syringe with 30-gauge needle [7].

**RNA scope *in situ* hybridization**

Mice were deeply anesthetized with an intraperitoneal injection of pentobarbital and transcardially perfused with phosphate-buffered saline (PBS) followed by ice-cold 4% paraformaldehyde/PBS. The transverse L4 segment of the spinal cord was quickly removed and postfixed in the same fixative overnight at 4°C. After incubation with 10%, 20%, and 30% sucrose solutions at 4°C, the tissues were embedded in OCT compound (Sakura Finetek Japan) and stored at -25°C before use. The L4 segment was sectioned at a thickness of 14 μm. *In situ* hybridization were performed using RNAscope^®^ Multiplex Fluorescent Reagent Kit v2 (ACDbio, 323100), according to the manufacturer’s instructions for fixed frozen tissues. The following probes were used: Mm-Adra1a (ACDbio, 408611), and Mm-Slc32a1-C3 (ACDbio, 319191-C3). Spinal cord sections were analyzed using an LSM700 Imaging System (ZEN 2012, Carl Zeiss). Cells were considered positive if three or more punctate dots were present in the nucleus and/or cytoplasm [8].

**Statistical analysis**

Statistical analyses were performed using Prism 7 (GraphPad). All data are shown as the mean ± SEM. Statistical significance of differences was determined using two-way repeated measures analysis of variance (ANOVA) with Bonferroni's multiple comparisons test (Fig. 1a, b, c, and e), one way ANOVA with Holm-Sidak’s multiple comparisons test (Fig. 1a, and b), Kruskal-Wallis test post hoc Dunn’s multiple comparisons test (Fig. 1a, and b), paired *t*-test (Fig.1c, and e), Wilcoxon matched-pairs signed rank test (Fig. 1c, and e), Mann-Whitney test (Fig. 1d). Differences were considered significant at *P* < 0.05.

**References**

1. Vong L, Ye C, Yang Z, Choi B, Chua S, Lowell BB. Leptin action on GABAergic neurons prevents obesity and reduces inhibitory tone to POMC neurons. Neuron. 2011;71(1):142-54.
2. Kohro Y, Matsuda T, Yoshihara K, Kohno K, Koga K, Katsuragi R, et al. Spinal astrocytes in superficial laminae gate brainstem descending control of mechanosensory hypersensitivity. Nat Neurosci. 2020;23(11):1376-87.
3. Atwal N, Winters BL, Vaughan CW. Endogenous cannabinoid modulation of restraint stress-induced analgesia in thermal nociception. J Neurochem. 2020;152(1):92-102.
4. Koga K, Yamagata R, Kohno K, Yamane T, Shiratori-Hayashi M, Kohro Y, et al. Sensitization of spinal itch transmission neurons in a mouse model of chronic itch requires an astrocytic factor. J Allergy Clin Immunol. 2020;145(1):183-91.e10.
5. Jonsson G, Hallman H, Ponzio F, Ross S. DSP4 (N-(2-chloroethyl)-N-ethyl-2-bromobenzylamine)--a useful denervation tool for central and peripheral noradrenaline neurons. Eur J Pharmacol. 1981;72(2-3):173-88.
6. Lyons WE, Fritschy JM, Grzanna R. The noradrenergic neurotoxin DSP-4 eliminates the coeruleospinal projection but spares projections of the A5 and A7 groups to the ventral horn of the rat spinal cord. J Neurosci. 1989;9(5):1481-9.
7. Hylden JL, Wilcox GL. Intrathecal morphine in mice: a new technique. Eur J Pharmacol. 1980;67(2-3):313-6.
8. Shiraishi Y, Koga K, Yamagata R, Hatada I, Shiratori-Hayashi M, Tsuda M. α_1A_-adrenaline receptors in dorsal horn inhibitory neurons have an inhibitory role in the regulation of chloroquine-induced itch in mice. Mol Brain. 2021;14(1):55.
