# Supplementary material for: Stress-induced antinociception to noxious heat requires α1A-adrenaline receptors of spinal inhibitory neurons in mice
Source: Mol Brain. 2022 Jan 3;15:6. doi: 10.1186/s13041-021-00895-3 (PMC8721982; doi:10.1186/s13041-021-00895-3)
Supplement: Supplementary file 2 — Additional file 2: Supplementary Figure 1. [file 13041_2021_895_MOESM2_ESM.docx]

**Additional file 2**


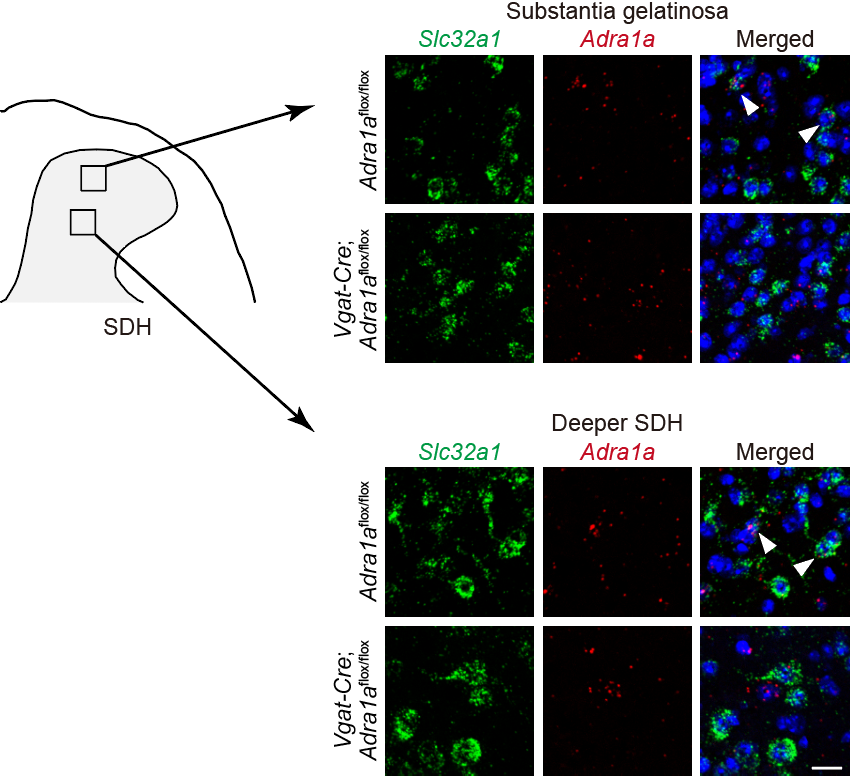


**Figure S1.**

**Fluorescence *in situ* hybridization of *Slc32a1* (*Vgat*) and *Adra1a* mRNAs in the SDH.**

RNAscope *in situ* hybridization for mRNAs for *Slc32a1* (green) and *Adra1a* (red) in the substantia gelatinosa (top) and the deeper SDH (bottom) of *Adra1a*^flox/flox^ mice and *Vgat-Cre*;*Adra1a*^flox/flox^ mice. 4,6-diamidino-2-phenylindole (DAPI) to mark nuclei is blue. Arrowheads indicate *Slc32a1*^+^*Adra1a*^+^ neurons. Scale bar: 20 μm.
